# AI-Based Brain Volumetry Without MPRAGE? Evaluation of Synthetic T1-MPRAGE from 2D T2/FLAIR †

**DOI:** 10.3390/diagnostics16020317

**Published:** 2026-01-19

**Authors:** Ludwig Singer, Tim Alexius Möhle, Angelika Mennecke, Konstantin Huhn, Veit Rothhammer, Manuel Alexander Schmidt, Arnd Doerfler, Stefan Lang

**Affiliations:** 1 Institute of Neuroradiology, University Hospital Erlangen, Friedrich-Alexander-University Erlangen-Nürnberg, 91054 Erlangen, Germany; 2 Department of Neurology, University Hospital Erlangen, Friedrich-Alexander-University Erlangen-Nürnberg, 91054 Erlangen, Germany

**Keywords:** brain volumetry, synthetic MRI, neurodegenerative disease imaging, brain atrophy assessment, artificial intelligence

## Abstract

**Background**: Automated AI-based brain volumetry is increasingly used in clinical practice. T1-weighted sequences (e.g., MPRAGE) are considered the current state-of-the art. However, due to faster acquisition and higher in-plane resolution, 2D anisotropic sequences are often preferred in clinical routine. However, these sequences cannot be processed with currently available AI-volumetry software. Thus, we here aimed to evaluate volumetric data from synthetic MPRAGE-like sequences (mprAIge). **Methods**: We analyzed 412 datasets (206 conventional MPRAGE and 206 T2w/FLAIR) from healthy volunteers (*n* = 36) and patients with multiple sclerosis (*n* = 140). Synthetic mprAIge was generated using SynthSR-CNN and assessed via assemblyNET on the volBrain platform. Total brain volume (TBV), gray and white matter volume (GMV/WMV), and key substructures were compared between mprAIge and conventional MPRAGE. Average volume differences (AVDs) and correlations were calculated. **Results**: Synthetic mprAIge was generated successfully in all 206 cases. Quantitative analysis demonstrated strong correlation and high agreement for key substructures. TBV showed excellent agreement (AVD: 2.75% for controls, 3.90% for MS patients; r = 0.99 and 0.97, respectively). White matter volume exhibited excellent agreement (AVD: −1.92% for controls, 0.28% for MS patients; r = 0.95). Hippocampal volume also demonstrated good to excellent agreement (AVD: 1.13% for controls, −1.92% for MS patients; r = 0.91 and 0.89, respectively). **Conclusions**: Synthetic mprAIge enables AI-volumetry software application without limitations. Its volumetric assessments align well with conventional MPRAGE, opening new opportunities for volumetric post-processing and mapping of disease progression.

## 1. Introduction

Magnetic Resonance Imaging (MRI) is frequently used in routine neuroimaging and serves across the entire spectrum of neurological conditions, from acute brain injuries to chronic neurodegenerative diseases. In recent years, and with the rise of artificial intelligence (AI) in medicine, there has been a significant increase in the use of automated post-processing methods, especially in inflammatory and degenerative diseases [1,2]. In particular, quantitative brain volumetry has gained increased clinical importance in the detection and follow-up of neurodegenerative diseases such as Alzheimer’s disease (AD) or in neuroinflammatory diseases such as multiple sclerosis (MS) [3]. Especially in the context of the newly approved disease-modifying anti-amyloid therapies, a uniform and automated volumetric assessment is essential to provide consistent monitoring over time [4,5]. Equally important is the assessment of brain volume loss in patients with MS, as this represents a key contributor to disability progression [6,7].

To achieve the best results and to minimize errors, high-resolution isotropic sequences, often a T1-MPRAGE (T1-weighted 3D magnetization-prepared rapid gradient echo) pulse sequence, are considered the current standard sequence used for many downstream analyses [8,9,10]. It is preferred for post-processing, as it provides detailed, isotropic volumetric images for accurate anatomical mapping and quantification [10,11,12].

However, conventional MPRAGE has a lengthy acquisition time and is prone to artifacts, e.g., due to motion, rendering the quality of the scan insufficient or even non-diagnostic for further analysis. While MPRAGE sequences remain the gold standard for volumetric analysis, an MPRAGE sequence is commonly not routinely acquired, instead the majority of scans are acquired as 2D, anisotropic images with a high in-plane resolution [8]. Additionally, the acquisition time is usually much shorter, making them less susceptible to motion artifacts.

This trade-off between acquisition time and image quality often necessitates compromises in clinical workflows, ultimately precluding many patients from high-quality quantitative and morphometrical analysis of the brain.

To address these limitations and to make legacy data accessible for high-resolution morphometry, multiple solutions have been proposed. Recent advances in artificial intelligence, deep learning, and convolutional neural networks (CNNs) are producing outstanding results in super-resolution and contrast synthesis of MRI [13,14,15,16]. One of these approaches is SynthSR from Iglesias et al., allowing us to generate isotropic images from a reference MR using a set of anisotropic 2D scans [13].

In this analysis, we used the SynthSR-CNN to generate isotropic, 1 mm MPRAGE-like images from anisotropic T2/FLAIR scans and evaluated its reliability against traditional MPRAGE in both healthy individuals and patients with MS. We hypothesize that (i) synthetic mprAIge enables automated brain volumetry from routine 2D sequences previously incompatible with AI-based brain volumetry software and (ii) volumetric measurements derived from mprAIge would demonstrate strong agreement with conventional 3D T1 MPRAGE. Utilizing 206 datasets (36 from healthy volunteers, 170 from patients with MS), this research seeks to validate these hypotheses by comparing synthetic MPRAGE (mprAIge) volumetric measurements against conventional MPRAGE to assess the consistency in both healthy controls and patients with MS. This would enable retrospective volumetric analysis of a large set of studies previously inaccessible for automated brain volumetry.

## 2. Materials and Methods

### 2.1. Study Population and Magnetic Resonance Imaging

To validate the accuracy of the SynthSR algorithm, we enrolled 36 healthy individuals (20 females; median age: 24 years, IQR 5.25) who underwent MRI on a 3 Tesla Siemens Magnetom Vida (Siemens Healthineers, Erlangen, Germany) at our institution. During a single imaging session, we acquired a 3D T1-weighted MPRAGE with 1 mm slice thickness, 2D T2-weighted Turbo Spin Echo (TSE) with 3 mm slice thickness, and 2D T2-weighted Fluid-Attenuated Inversion Recovery (FLAIR) with 3 mm slice thickness. Recruitment was stopped after 36 individuals, as preliminary analysis demonstrated minimal differences in brain volumes between MPRAGE and mprAIge.

**Figure 1:**
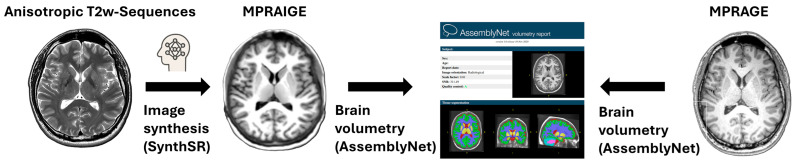
Schematic workflow of synthetic MPRAIGE generation and volumetric evaluation. Anisotropic 2D T2-w/FLAIR sequences are processed using pretrained SynthSR CNN to generate synthetic T1w MPRAIGE. Both conventional and synthetic MPRAGE are then analyzed using the AssemblyNet framework for automated whole-brain volumetry.

Additionally, we analyzed 170 clinical MRI scans from patients with multiple sclerosis (MS) (*n* = 140, 117 females; median age: 57 years, IQR 7), acquired either on a 1.5 Tesla Siemens Magnetom Sola.Fit or a 3 Tesla Siemens Magnetom Vida during routine follow-up in our institution between January 2023 and December 2024. Inclusion criteria for this cohort were over 50 years and presence of at least five white matter hyperintensities (WMH), targeting individuals with long-standing disease progression. This cohort allowed a more robust evaluation of SynthSR performance in the presence of marked structural changes. All patients underwent the established routine clinical protocol, which includes a 3D T1-MPRAGE and a 2D T2 TSE sequence used for generating the synthetic mprAIge. Eleven datasets were excluded due to excessive motion artifacts. Detailed scan parameters are provided in Appendix A. A flow chart of the analysis performed is provided in Figure 1.

### 2.2. Image Analysis, Data Preparation, and Artificial Intelligence (AI)-Based Volumetry

**Figure 2:**
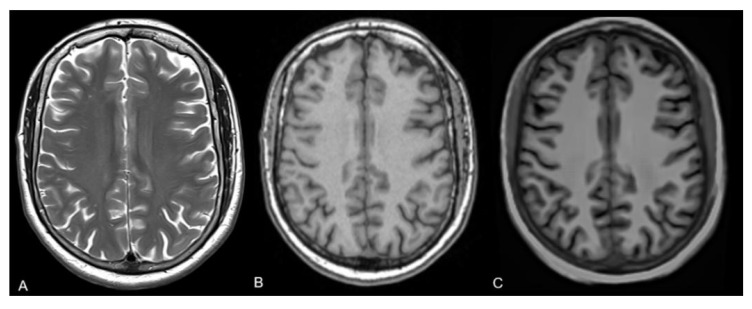
Example images of (**A**) 2D T2 TSE, (**B**) 3D T1-MPRAGE, and (**C**) synthetic 3D T1-MPRAGE in axial orientation.

The datasets were exported and then converted into mprAIges following the publicly available Synth-SR algorithm (https://github.com/BBillot/SynthSR; accessed on 12 December 2025) [13]. This CNN was trained on twenty 1 mm isotropic 3D MPRAGE scans from the Open Access Series of Imaging Studies (OASIS) dataset. Extracerebral regions were automatically obtained with an atlas-based Bayesian approach, including eyes, skull, and soft tissue [9,17,18]. Furthermore, a two-layer U-net architecture is used, while one layer is responsible for regression and the other for segmentation. The regression CNN predicts high-resolution intensities from synthetic low-resolution inputs generated via a Gaussian Mixture Model (GMM), while the other performs segmentation of the dataset, ensuring that the images are anatomically accurate [13]. An example is given in Figure 2.

**Figure 3:**
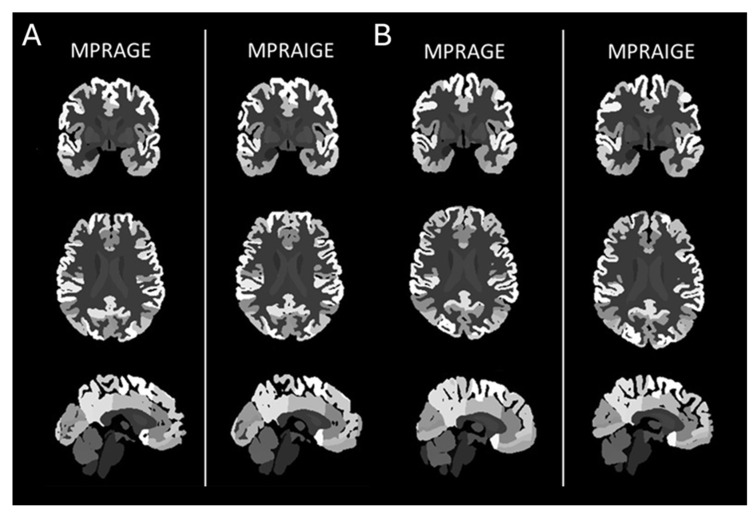
Example segmentation outputs of T1-MPRAGE (left) and mprAIge (right) with coronal, axial, and sagittal orientation. (**A**,**B**) Segmentations closely resemble each other, demonstrating consistent representation of major anatomical structures. Notably, the parietal and occipital lobes are well preserved across both modalities, while the frontal lobe in the synthetic image appears slightly less defined.

After successful conversion, real and synthetic MPRAGE were evaluated using AssemblyNet (https://github.com/volBrain/AssemblyNet; accessed on 3 November 2025), an advanced deep learning framework designed for whole-brain segmentation and volumetry [19,20]. Compared to conventional clinically available deep learning U-Net models, it uses an “assembly” of 250 U-Nets in two assemblies. The first assembly generates an initial brain segmentation at 2 × 2 × 2 mm^3^ resolution. This results in a basic structural overview of the brain, identifying major anatomical regions with a coarse but comprehensive approach. The second assembly refines the decision taken by the first one at a higher resolution, and a final decision is obtained by majority voting [21]. A comparison of segmentation is given in Figure 3.

Statistical analysis was performed using Python 3.12 and R 4.4.0 [22,23]. Average volume differences (AVDs), volume correlations using Pearson correlations (r), and root mean square error (RMSE) were assessed. Normality of distribution was assessed using the Shapiro–Wilk test. For pairwise comparisons, a paired *t*-test was used for normally distributed data; otherwise, the Wilcoxon signed-rank test was applied. The threshold of statistical significance was defined as *p* < 0.05.

## 3. Results

### 3.1. Generation of Synthetic mprAIge

Synthetic mprAIge was successfully generated in all 206 individual scans. Quantitative analysis revealed good to excellent correlation between MPRAGE and mprAIge volumes across different brain regions. Table 1 and Table 2.

### 3.2. Healthy Control Group

#### 3.2.1. Global Volumetric Analysis

In the volumetric analysis of the control group, synthetic mprAIge yielded higher total brain volume measurements compared to conventional MPRAGE (1328.92 mL vs. 1298.70 mL), at an average volume difference (AVD) of 2.33%, with an excellent correlation (r = 0.987) and a root mean square error (RMSE) of 40.75 mL. White matter volume was slightly lower in the synthetic data (482.52 mL vs. 493.88 mL; AVD: −2.30%, r = 0.953, RMSE = 17.01 mL), while gray matter volume was higher (846.42 mL vs. 804.79 mL; AVD: 5.17%, r = 0.985, RMSE = 47.28 mL).

#### 3.2.2. Regional Substructure Analysis

Measurements of the frontal lobe yielded higher volumes in the synthetic mprAIge compared to the conventional scan (225.89 mL vs. 211.46 mL; AVD: 6.82%, r = 0.959, RMSE = 15.58 mL). Temporal lobe volume was also higher in the synthetic data (135.36 mL vs. 126.06 mL; AVD: 7.38%, r = 0.972, RMSE = 10.33 mL). Consistently increased volumes were observed in the parietal lobe (131.18 mL vs. 125.69 mL; AVD: 4.37%, r = 0.973, RMSE = 6.55 mL), occipital lobe (96.74 mL vs. 93.06 mL; AVD: 3.96%, r = 0.983, RMSE = 5.09 mL), and limbic cortex (48.01 mL vs. 46.30 mL; AVD: 3.67%, r = 0.979, RMSE = 2.27 mL). The cerebellum also yielded higher volumes in the mprAIge (141.76 mL vs. 137.13 mL), with an AVD of 3.37%, r = 0.891, and RMSE = 9.22 mL.

#### 3.2.3. Subcortical Volumetric Analysis

The hippocampi exhibited slightly higher volumes in the synthetic mprAIge compared to the conventional scan (7.70 mL vs. 7.57 mL; AVD: 1.69%, r = 0.912, RMSE = 0.28 mL). The amygdalae also yielded higher volumes (2.37 mL vs. 2.27 mL; AVD: 4.70%, r = 0.725, RMSE = 0.31 mL). In contrast, the brainstem volume was lower in the synthetic data (18.32 mL vs. 18.82 mL), corresponding to an AVD of −2.66%, r = 0.966, and RMSE = 0.81 mL.

### 3.3. MS Group

#### 3.3.1. Global Volumetric Analysis

In the volumetric analysis of the MS group, synthetic mprAIge produced higher total brain volumes compared to conventional MPRAGE (1195.17 mL vs. 1149.22 mL), corresponding to an average volume difference (AVD) of 3.90%, with excellent correlation (r = 0.97) and RMSE = 53.31 mL. White matter volume was also higher in the synthetic data (426.53 mL vs. 424.73 mL; AVD: 0.28%, r = 0.94, RMSE = 18.49 mL), while maintaining excellent correlation. Gray matter volume measurements also resulted in higher results with excellent correlations (768.07 mL vs. 724.95 mL; AVD: 6.02%, r = 0.98, RMSE = 46.88 mL).

#### 3.3.2. Regional Substructure Analysis

Frontal lobe volume was higher in the synthetic mprAIge (199.06 mL vs. 186.55 mL; AVD: 7.37%, r = 0.98, RMSE = 14.70 mL), as was temporal lobe volume (123.17 mL vs. 116.08 mL; AVD: 6.66%, r = 0.94, RMSE = 8.91 mL). Consistently increased volumes were observed in the parietal lobe (115.41 mL vs. 110.45 mL; AVD: 3.59%, r = 0.98, RMSE = 4.92 mL), occipital lobe (92.17 mL vs. 87.72 mL; AVD: 5.12%, r = 0.96, RMSE = 5.50 mL), and limbic cortex (43.96 mL vs. 42.24 mL; AVD: 3.70%, r = 0.95, RMSE = 2.40 mL). The cerebellum also showed increased volume in the mprAIge (136.27 mL vs. 123.75 mL), with an AVD of 10.35%, r = 0.86, and RMSE = 14.18 mL.

#### 3.3.3. Subcortical Volumetric Analysis

The hippocampi yielded lower volumes in the synthetic mprAIge with good correlations (6.90 mL vs. 7.12 mL; AVD: −1.92%, r = 0.89, RMSE = 0.41 mL). The amygdalae demonstrated increased volumes (2.22 mL vs. 2.16 mL; AVD: 2.09%, r = 0.88, RMSE = 0.14 mL). Brainstem volume was lower in the synthetic data (16.88 mL vs. 17.55 mL; AVD: −3.68%, r = 0.98, RMSE = 0.83 mL).

**Figure 4:**
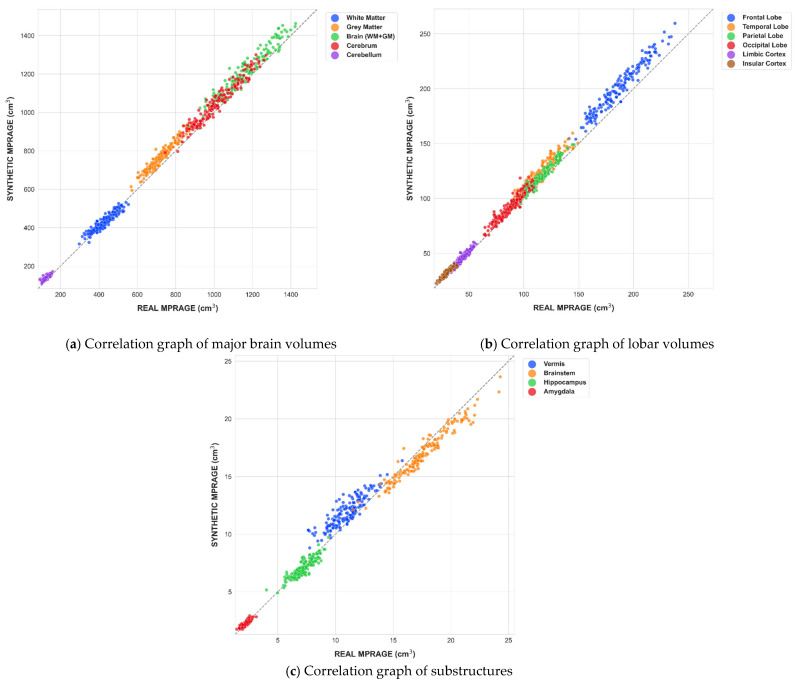
Scatter plots illustrating the relationship between real MPRAGE and synthetic MPRAGE volumetric estimates across multiple brain regions.

A detailed summary of all correlations is shown in Figure 4.

### 3.4. Statistical Testing

#### 3.4.1. Control Group

Significant differences were observed in global volumetric measures, including total brain volume (TBV), gray matter volume (GMV), and white matter volume (WMV) (all *p* < 0.001). Regional brain structure analysis showed statistically significant differences in the frontal, temporal, parietal, and occipital lobes, as well as the limbic cortex and cerebellum (all *p* < 0.001). Among subcortical structures, the amygdala (*p* = 0.0018) and brainstem (*p* < 0.001) exhibited significant differences, whereas the volume differences in the hippocampus did not reach statistical significance (*p* = 0.0686).

#### 3.4.2. MS-Patient Group

Similar to the overall findings, significant differences were observed in global volumetric measures, including TBV and GMV (both *p* < 0.001), whereas WMV did not show a significant difference (*p* = 0.453). Regional analysis revealed significant differences across the frontal, temporal, parietal, and occipital lobes, as well as the limbic cortex and cerebellum (all *p* < 0.001). In subcortical structures, the hippocampus, amygdala, and brainstem all showed statistically significant differences (all *p* < 0.001).

## 4. Discussion

In recent years, artificial intelligence has been on the rise in medicine, especially in the radiological field. One integral part is tools for brain segmentation and brain volumetry, for example, in the diagnosis or follow-up of neurodegenerative diseases such as Alzheimer’s disease or neuroinflammatory diseases such as multiple sclerosis [20,24,25]. Moreover, during longitudinal assessment of neuroinflammatory and neurodegenerative diseases, especially in light of the introduction of disease-modifying anti-amyloid therapies, a uniform, rater-independent, and repeatable volumetric assessment to monitor disease progression and therapy response across an entire patient population is necessary.

For these evaluations, T1-weighted MPRAGE sequences have been considered the gold standard, yet in routine clinical imaging, anisotropic 2D sequences are more commonly used. These are often incompatible with AI-based brain volumetry tools. Addressing this limitation, we investigated the practicality and validity of synthetically generating T1-MPRAGEs (mprAIge) from anisotropic routine FLAIR/T2 scans, enabling high-quality volumetric evaluations for previously inaccessible neuroimaging data.

We hypothesized that synthetic mprAIge allows automated brain volumetry from scans previously inaccessible and that volumetric measurements derived from these sequences show strong agreement with conventional 3D T1-MPRAGE, both in healthy individuals and in patients with multiple sclerosis.

Our findings support both hypotheses. Synthetic image generation was successful in all 206 individual scans, demonstrating technical feasibility as an “out of the box” solution. Volumetry resulted in good to excellent correlations with only minor volume differences across different brain structures in our patient population. In healthy individuals, synthetic mprAIge demonstrated excellent correlations across various brain regions. Total brain volume yielded higher results, while maintaining excellent matter volumes showed a modest overestimation (AVD = 5.17%, r = 0.985), while white matter volumes exhibited a small underestimation (AVD = −2.30%, r = 0.953). Subcortical structures, including the hippocampi and amygdalae, demonstrated minimal differences and maintained strong correlations (r > 0.89).

To further evaluate the synthetic mprAIge, we chose patients with multiple sclerosis (MS) above the age of 50 with at least five white matter lesions as our patient group. Furthermore, MS patients often exhibit structural pathologies, including white matter hyperintensities and atrophy, providing an interesting context to test the robustness and accuracy of the synthetic mprAIge method.

Total brain volume was also slightly overestimated in the patient group (AVD = 3.90%, r = 0.97, RMSE = 53.31 mL), with gray matter volumes showing a small increase (AVD = 6.02%, r = 0.98) and white matter volumes remaining stable (AVD = 0.28%, r = 0.94). The frontal and temporal lobes displayed the largest discrepancies (7.37% and 6.66% overestimation, respectively). These variations are likely attributable to the anisotropic resolution and the complex gyral architecture of these regions, where 3 mm slice thickness may inadequately delineate boundaries. The occipital and parietal lobes exhibited smaller differences, with increases of 5.12% and 3.59%, respectively. Subcortical volumes such as the hippocampi and amygdalae varied slightly (−1.92% and +2.09%, respectively) but maintained high correlations (r > 0.89).

These findings demonstrate that synthetic mprAIge performs reliably even in the presence of disease-induced structural changes, such as the presence of white matter lesions or generalized atrophy. This method offers an easy, out-of-the-box solution with minimal preprocessing requirements, making it highly accessible for both research and clinical applications. For total brain volume, gray matter, and white matter, the synthetic method provides reliable estimates. While discrepancies were more pronounced in the frontal, temporal, and cerebellar regions, likely reflecting the difficulty of accurately delineating intricate gyral or infratentorial structures in anisotropic data, these systematic differences were consistent across subjects and primarily attributable to the limitations of the source resolution rather than random error. Importantly, for clinical applications focused on overall brain volume or global atrophy assessment, these regional deviations are minor and do not substantially affect interpretability. Less structurally complex regions, such as the occipital and parietal lobes, exhibited smaller and more uniform differences, reinforcing the method’s dependability in capturing general anatomical features.

**Figure 5:**
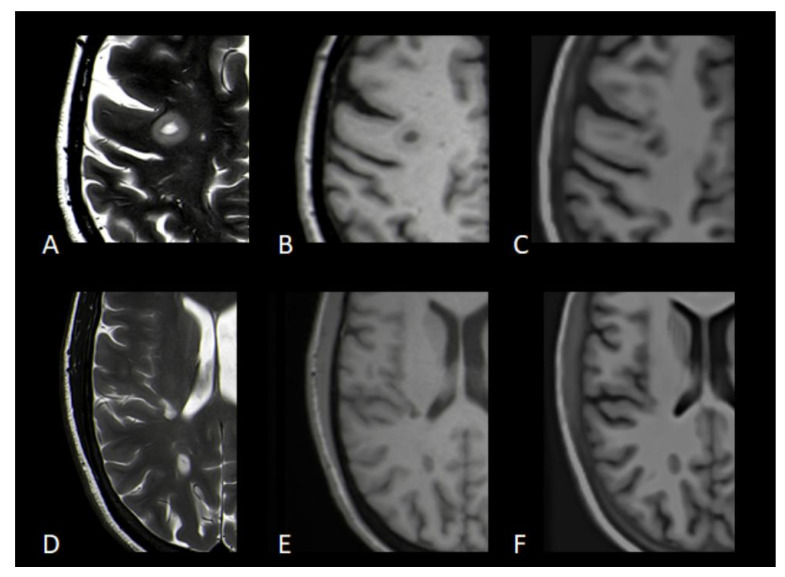
Representative images of a 55-year-old male patient with MS: (**A**) 2D T2 TSE, (**B**) 3D T1-MPRAGE, and (**C**) synthetic 3D T1-MPRAGE; a 55-year-old female patient with MS: (**D**) 2D T2 TSE, (**E**) 3D T1-MPRAGE, and (**F**) synthetic 3D T1-MPRAGE. Lesions in the right frontal and right occipital lobes are visible in both the original and mprAIge images. However, these lesions exhibit changes in geometry, and a lesion adjacent to the right lateral ventricle seen in (**D**) is not apparent in the corresponding synthetic image (**F**).

Although substructures like the hippocampi and amygdalae showed high correlations, the observed variation in these smaller regions suggests limitations in the synthetic MPRAGE’s ability to precisely measure finer anatomical details, indicating that while synthetic mprAIge is an excellent tool for obtaining a general overview of brain structure, it may not yet replace traditional imaging for studies requiring precise quantification of substructures or fine anatomical features. Another important observation in the MS patient group was the reproducibility of white matter lesions from T2w sequences into the synthetic mprAIge images. While lesion analysis on synthetic mprAIge was not a primary focus of this study, it carries significant clinical implications, particularly for the MS patient group. In our dataset, larger lesions, particularly confluent periventricular and deep white matter hyperintensities, were well represented in synthetic images. However, small lesions, especially juxtacortical, periventricular “dot-like” lesions, frequently showed altered lesion geometry in the synthetic reconstructions; see Figure 5.

Additionally, the ability to generate synthetic MPRAGE-like images from routine 2D T2/FLAIR sequences also has major implications in the management and follow-up of neurodegenerative diseases. With the recent approval of anti-amyloid therapies such as Lecanemab and Donanemab for Alzheimer’s disease, there is great clinical interest in having a standardized and reproducible volumetric assessment to establish baseline measurements or to visualize changes over time. In these settings, mprAIge may serve as a substitution for conventional MPRAGE when 3D T1-weighted imaging is not available. Our approach enables AI-based volumetry from previously acquired imaging sessions where a T1-MPRAGE might be missing, enabling legacy data for automated brain volumetry. This retrospective capability allows the establishment of a baseline volumetric measurement and the visualization of changes over time, which is particularly interesting for patients who may be eligible for anti-amyloid therapies but whose previous scan lacked isotropic 3D T1 sequences.

In summary, synthetic mprAIge demonstrates low percentual deviations and high correlations with standard MPRAGE, establishing its reliability for neuroimaging applications. It provides reliable volumetric data for total brain volume, gray matter, and white matter, even in populations with structural changes such as multiple sclerosis. While some discrepancies are more pronounced in regions with complex gyral anatomy, such as the frontal and temporal lobes, the overall performance remains consistent and robust.

While these findings indicate that synthetic mprAIge is not a full substitute for conventional 3D T1-MRPAGE, it enables automated volumetric analysis of legacy data and routine MRI datasets through AI-based image synthesis previously inaccessible to these evaluations. Further refinement is necessary to improve the precision of smaller substructures, such as the hippocampi and amygdalae.

## 5. Limitations

We certainly acknowledge the limitations of these analyses. The anisotropic 2D T2/FLAIR sequences utilized in this study had a slice thickness of 3 mm, which might not be the general standard, as slice thicknesses of 4–5 mm are more frequently used. This higher resolution may have contributed to improved synthetic image generation and volumetric accuracy, limiting the generalizability of our findings to datasets with lower spatial resolution.

Secondly, our analysis was conducted using only one type of volumetric software. While this ensures consistency, it also restricts the generalizability of this analysis, as results may vary with different segmentation algorithms. AssemblyNet was chosen because it is publicly available and has demonstrated high performance compared with single U-Net–based approaches.

Additionally, the synthetic images were generated using T2/FLAIR sequences alone; we did not test conversions from T1-weighted or other sequence types, orientations, or contrast weightings, which may offer complementary information or yield different reconstruction characteristics. While synthetic mprAIge demonstrated reliable performance for global and lobar volumes, the quantification of smaller substructures, such as the hippocampi and amygdalae, remains less precise. This limitation highlights the need for further optimization of synthetic imaging techniques to improve their ability to capture fine anatomical details.

Another limitation lies in the depiction of pathological features. In synthetic mprAIge images, larger lesions, such as confluent periventricular or deep white matter hyperintensities, were generally well preserved, while smaller lesions were often distorted or entirely absent (Figure 5). This suggests that, while synthetic mprAIge can approximate anatomical structures with good accuracy, it cannot yet serve as a full replacement for conventional MPRAGE in all diagnostic contexts.

## 6. Conclusions

Our analysis demonstrates the practicality and validity of synthetic mprAIge for brain volumetry, providing robust estimates of total brain volume, gray and white matter, and key substructures such as the hippocampi and amygdalae. This approach enables volumetric analysis of routine anisotropic T2/FLAIR scans, overcoming limitations of AI-based volumetry tools requiring 3D-T1 sequences. These findings highlight its potential as a practical and accessible tool for both research and clinical neuroimaging, with room for further refinement in capturing finer anatomical details. Importantly, in light of the recently approved anti-amyloid treatment in Alzheimer’s disease, this method could enable an out-of-the-box volumetric analysis from existing anisotropic scans, generating a baseline for treatment monitoring and follow-up. 

## Figures and Tables

**Table 1:** Comparison of volumetric measurements between synthetic mprAIge and conventional MPRAGE in the healthy control group. Values are reported as means and standard deviations (SDs). Pearson correlation coefficients (r), root mean square errors (RMSEs), and percentage differences are provided to assess agreement between modalities.

Region	MPRAGE (Mean ± SD)	mprAIge (Mean ± SD)	r	RMSE	Diff. %
White Matter (WM) volume (cm^3^)	492.16 ± 46.45	493.04 ± 44.69	0.95	17.01	−1.92
Grey Matter (GM) volume (cm^3^)	807.18 ± 69.50	857.09 ± 75.20	0.98	47.28	5.62
Brain (WM+GM) volume (cm^3^)	1298.68 ± 113.03	1350.90 ± 119.44	0.99	40.75	2.75
Cerebrum total volume (cm^3^)	1146.55 ± 102.69	1191.73 ± 108.39	0.98	34.63	2.46
Cerebellum total volume (cm^3^)	135.64 ± 12.73	145.08 ± 12.71	0.89	9.22	5.14
Vermis volume (cm^3^)	12.34 ± 1.28	12.64 ± 1.24	0.92	0.64	3.21
Brainstem volume (cm^3^)	18.91 ± 1.92	18.08 ± 2.00	0.97	0.81	−3.32
Frontal lobe total volume (cm^3^)	210.20 ± 19.81	224.66 ± 21.46	0.96	15.58	6.78
Temporal lobe total volume (cm^3^)	125.18 ± 12.58	137.36 ± 13.35	0.97	10.33	7.81
Parietal lobe total volume (cm^3^)	124.62 ± 12.89	131.18 ± 13.60	0.97	6.55	4.57
Occipital lobe total volume (cm^3^)	93.25 ± 8.26	97.35 ± 9.76	0.98	5.09	4.92
Limbic cortex total volume (cm^3^)	45.46 ± 4.86	47.16 ± 5.45	0.98	2.27	4.15
Insular cortex total volume (cm^3^)	32.46 ± 3.69	34.72 ± 3.63	0.96	2.01	5.36
Hippocampus total volume (cm^3^)	7.67 ± 0.63	7.80 ± 0.65	0.91	0.28	1.13
Amygdala total volume (cm^3^)	2.27 ± 0.40	2.37 ± 0.20	0.73	0.31	4.70

**Table 2:** Comparison of volumetric measurements between synthetic mprAIge and conventional MPRAGE in the MS-patient group. Values are reported as means and standard deviations (SDs). Pearson correlation coefficients (r), root mean square errors (RMSEs), and percentage differences are provided to assess agreement between modalities.

Region	MPRAGE (Mean ± SD)	mprAIge (Mean ± SD)	r	RMSE	Diff. %
White Matter (WM) volume (cm^3^)	424.73 ± 52.58	426.53 ± 45.97	0.94	18.49	0.28
Grey Matter (GM) volume (cm^3^)	724.95 ± 70.70	768.07 ± 75.38	0.98	46.88	6.02
Brain (WM+GM) volume (cm^3^)	1149.22 ± 119.30	1195.17 ± 120.52	0.97	53.31	3.90
Cerebrum total volume (cm^3^)	1009.52 ± 109.90	1045.58 ± 111.50	0.98	40.00	3.07
Cerebellum total volume (cm^3^)	123.75 ± 11.48	136.27 ± 11.18	0.86	14.18	10.35
Vermis volume (cm^3^)	11.03 ± 1.38	11.74 ± 1.29	0.89	1.13	8.38
Brainstem volume (cm^3^)	17.55 ± 2.26	16.88 ± 2.09	0.98	0.83	−3.68
Frontal lobe total volume (cm^3^)	186.55 ± 20.34	199.06 ± 22.01	0.98	14.70	7.37
Temporal lobe total volume (cm^3^)	116.08 ± 12.39	123.17 ± 12.86	0.94	8.91	6.66
Parietal lobe total volume (cm^3^)	110.45 ± 12.75	115.41 ± 13.50	0.98	4.92	3.59
Occipital lobe total volume (cm^3^)	87.72 ± 10.16	92.17 ± 10.84	0.96	5.50	5.12
Limbic cortex total volume (cm^3^)	42.24 ± 5.17	43.96 ± 5.69	0.95	2.40	3.70
Insular cortex total volume (cm^3^)	29.46 ± 3.45	31.77 ± 3.53	0.94	2.56	7.49
Hippocampus total volume (cm^3^)	7.12 ± 0.85	6.90 ± 0.78	0.89	0.41	−1.92
Amygdala total volume (cm^3^)	2.16 ± 0.28	2.22 ± 0.25	0.88	0.14	2.09

## Data Availability

The data presented in this study are available on request from the corresponding author due to legal and ethical reasons.

## Abbreviations

AD	Alzheimer’s disease
AI	Artificial Intelligence
AVD	Average Volume Difference
CNN	Convolutional Neural Network
FLAIR	Fluid-Attenuated Inversion Recovery
GM	Gray Matter
GMV	Gray Matter Volume
IQR	Interquartile Range
MPRAGE	Magnetization-Prepared Rapid Gradient Echo
MRI	Magnetic Resonance Imaging
MS	Multiple Sclerosis
OASIS	Open Access Series of Imaging Studies
RMSE	Root Mean Square Error
TBV	Total Brain Volume
TSE	Turbo Spin Echo
WM	White Matter
WMH	White Matter Hyperintensities
WMV	White Matter Volume

## Appendix A. Detailed Summary of Sequence Parameters

**Sequence**	**2D T2w (3.0T)**	**2D FLAIR (3.0T)**	**3D MPRAGE (3.0T)**	**2D T2w (1.5T)**	**3D MPRAGE (1.5T)**
Scanning Sequence	TSE	[TSE, IR]	[GR, IR]	TSE	[GR, IR]
Mode	2D	2D	3D	2D	3D
Slice Thickness (mm)	3.0	3.0	1.0	3.0	1.1
Repetition Time (ms)	6000	8000	2300	6000	1990
Echo Time (ms)	96	87	3.20	96	2.67
Inversion Time (ms)	None	2370	900	None	None
Acquisition Matrix	[0,320,256,0]	[0,320,256,0]	[0,256,256,0]	[0,320,256,0]	[0,256,256,0]
Imaging Frequency (MHz)	123.26	123.26	123.26	63.86	63.86
Duration (min:s)	1:21	1:33	5:12	1:16	4:30
